# Immune-related gene expression in severe periodontitis assessed by NanoString technology: A preliminary study

**DOI:** 10.17305/bb.2025.13313

**Published:** 2025-12-05

**Authors:** Dragomira Nikolova, Velitchka Dosseva-Panova, Dimitar Dimitrov, Savina Hadjidekova, Ivanka Dimova

**Affiliations:** 1 Department of Medical Genetics, Faculty of Medicine, Medical University of Sofia, Sofia, Bulgaria; 2 Department of Periodontology, Faculty of Dental Medicine, Medical University of Sofia, Sofia, Bulgaria

**Keywords:** Severe periodontitis, inflammation, gene expression, differentially expressed genes, DEGs, NanoString technology

## Abstract

Periodontitis is an inflammatory disease characterized by the destruction of the periodontal attachment apparatus, which includes alveolar bone, periodontal ligament, and cementum. This destruction is driven by a dysregulated host immune response to pathogenic subgingival biofilm. The present preliminary study aimed to evaluate immune-related gene expression patterns in patients with stage III/IV periodontitis utilizing the NanoString nCounter^®^ platform. Unstimulated saliva samples were collected from 12 individuals: ten with severe periodontitis (stage III/IV) and two periodontally healthy controls. Total RNA was isolated and analyzed using the nCounter^®^ Human Inflammation Panel, which profiles 249 inflammation-associated human genes. Data normalization and differential expression analysis were performed with nSolver™ software. Following quality control, genes with low expression (mean normalized counts < 20) were excluded, resulting in 89 genes available for comparison. Among these, 26 genes (29.2%) met a predefined effect-size threshold (|log_2_FC| ≥ 1), comprising 23 upregulated and 3 downregulated transcripts in the periodontitis group. Notably, the upregulated genes *HLA-DRB1* (*P* ═ 0.003; FDR = 0.267) and *CCR1* (*P* ═ 0.007; FDR = 0.312) exhibited relatively large log_2_ fold changes and the lowest unadjusted *P* values; however, neither retained significance after FDR correction. These findings underscore the feasibility of salivary gene expression profiling as a method for identifying molecular markers associated with disease severity. Given their roles in immune activation and leukocyte recruitment, *HLA-DRB1* and *CCR1* emerge as potential biomarker candidates for detection, risk stratification, and therapeutic monitoring in periodontitis, necessitating validation in larger, well-characterized cohorts.

## Introduction

Periodontitis is a complex, multifactorial disease influenced by environmental, behavioral, and genetic factors. Its development is characterized by alterations in the oral microbiome and dysregulation of the host immune response [1]. A hallmark of this dysregulation is persistent inflammation, which exacerbates clinical symptoms and contributes to the progressive destruction of the periodontal attachment apparatus. Beyond local tissue destruction, the systemic inflammatory burden associated with periodontitis has been linked to several systemic conditions, including cardiovascular diseases, diabetes mellitus, and adverse pregnancy outcomes.

Severe periodontitis affects approximately 7%–14% of the population in Europe and North America [2, 3]. Established risk factors such as poor oral hygiene, unhealthy nutrition, and extensive smoking are associated with disease progression; however, variables including ethnicity, systemic comorbidities, and genetic predisposition also significantly influence its global prevalence. Numerous studies have documented alterations in immune cell populations among individuals with periodontitis, such as increased neutrophil phagocytic activity potentially linked to polymorphisms in the NADPH oxidase gene [4], elevated presence of activated B-cells and plasma cells within periodontal lesions [1], and increased monocyte counts [5]. Periodontal inflammation is a central pathogenic feature, facilitating the progression from gingival involvement to alveolar bone resorption.

According to Schäfer et al. [6], the genetic contribution to periodontitis pathogenesis is estimated to range from approximately 25% in older individuals to up to 50% in younger patients. Nonetheless, the precise genetic factors involved remain incompletely understood and vary considerably among different populations, reflecting ethnic heterogeneity. Genome-wide association studies (GWAS) have identified single-nucleotide polymorphisms (SNPs) in 38 genes associated with periodontitis [7]. Various research efforts have sought to identify molecular biomarkers for the disease. For instance, Ban et al. employed bioinformatics analyses to identify 327 differentially expressed genes in periodontitis, including 149 upregulated and 178 downregulated genes, with 27 being immune-related. They highlighted two immune-related genes, IGNG and TLR2, as potential therapeutic targets [8]. Similarly, Cárdenas et al. [9] investigated epigenetic biomarkers by examining DNA methylation and RNA expression patterns, reporting hypermethylation in several Toll-like receptor (TLR)-related genes. Recently, Li et al. [10] utilized machine-learning approaches to identify seven immune-related genes as prospective targets for therapeutic intervention.

The hypothesis that periodontitis develops earlier or exhibits greater severity in genetically susceptible individuals underscores the necessity for further research to elucidate the molecular pathways involved. This study represents the first pilot investigation of immune-related gene expression profiles in Bulgarian patients with stage III/IV periodontitis.

To date, only a limited number of studies have explored the use of saliva for large-scale gene expression profiling in periodontitis. Investigating salivary RNA may provide new insights into the molecular mechanisms underlying disease development and progression and support the identification of potential diagnostic or prognostic markers.

The aim of this preliminary study was to assess the expression patterns of immune-related genes in saliva samples from patients with stage III/IV periodontitis using the NanoString nCounter^®^ Human Inflammation Panel. By mapping the expression of 249 inflammation-associated genes, this study seeks to contribute to the growing body of evidence regarding the molecular basis of immune dysregulation in severe periodontitis and to evaluate the feasibility of saliva-based transcriptomic analysis for future periodontal research.

## Materials and methods

### Selection criteria

Participants were recruited based on the following inclusion criteria: 1) adults aged 18–70 years; 2) diagnosis of Stage III/IV periodontitis according to the 2018 classification by the American Academy of Periodontology (AAP) and the European Federation of Periodontology (EFP) [11], defined by the presence of at least one of the following clinical and radiographic parameters:

- Interdental clinical attachment loss (CAL) ≥5 mm at ≥2 non-adjacent teeth;
- Radiographic bone loss extending to the middle or apical third of the root;
- Tooth loss due to periodontitis;
- Probing pocket depth (PPD) ≥6 mm at one or more sites;
- Vertical bone loss ≥3 mm at one or more sites;
- Furcation involvement classified as Class II or III.

The exclusion criteria were established as follows: 1) Use of systemic anti-inflammatory or immunosuppressive drugs, including corticosteroids, within the past six months; 2) Antibiotic use within the six months preceding sample collection; 3) Pregnancy or breastfeeding; 4) Receipt of periodontal therapy within the prior 12 months.

### Study groups

We collected approximately 2 mL of saliva samples from patients examined and treated at the Department of Periodontology, Faculty of Dental Medicine, Medical University of Sofia, Bulgaria, after a minimum fasting period of two hours. A total of 12 patients were analyzed—ten with stage III/IV periodontitis and two periodontally healthy individuals. The mean age of the patient group was 49.8 years (51.2 years for males; 48.4 years for females). The periodontal status of the patients was assessed by measuring the full mouth plaque score (FMPS), full mouth bleeding score (FMBS), PPD, CAL, bleeding on probing (BoP), and the bone loss to age ratio (BL/Age). Detailed clinical data for the study participants are presented in Table 1.

**Table 1:** Epidemiological and clinical data of study participants

**Personal number of the sample**	**Gender of the patient**	**Age at examination (years)**	**Stage of periodontitis**	**Smoking habits**	**FMPS (%)**	**FMBS (%)**	**PPD > 5 mm (%)**	**CAL ≥ 5 mm (%)**	**BoP (%)**	**BL/Age**
6	Male	49	III	>10 cigarettes daily	92	68	14	38	43	0.95
8	Female	50	III	>10 cigarettes daily	32	29	11	42	26	1.04
9	Female	55	III	no	68	62	22	37	64	1.09
12	Male	40	III	>10 cigarettes daily	87	43	6	56	26	1.15
13	Female	45	III	no	64	58	18	32	52	0.86
14	Female	45	III	no	26	43	16	52	47	1.23
17	Male	44	III	no	73	37	18	21	31	0.75
19	Male	61	III	no	45	36	8	41	34	0.65
22	Male	62	III	>10 cigarettes daily	84	74	12	28	69	0.78
23	Female	47	IV	no	76	59	7	23	63	0.89
24	Female	33	No periodontitis	<10 cigarettes daily	28	8	0	0	8	No BL
29	Male	29	No periodontitis	no	12	3	0	0	3	No BL

Ten individuals, comprising an equal number of males and females, exhibit severe periodontitis, while two individuals—a male and a female—are periodontally healthy. Abbreviations: FMPS: Full mouth plaque score; FMBS: Full mouth bleeding score; PPD: Probing pocket depth; CAL: Clinical attachment loss; BoP: Bleeding on probing; BL/Age: Bone loss to age ratio.

### RNA isolation and quality estimation

Saliva samples were stored in a preservation buffer (RNA/DNA shield, Zymo, CA, USA) until total RNA isolation (up to one week). For RNA isolation, we employed the Quick-RNA Miniprep Plus Kit (Zymo, CA, USA), which incorporates a sample preservation system combined with Zymo-Spin column technology and Proteinase K for optimal RNA extraction (catalog #R1057). In total, 12 RNA samples were isolated from the saliva of ten patients with stage III/IV periodontitis and two periodontally healthy controls. The quality and concentration of all RNA samples were assessed using a NanoDrop™ spectrophotometer (Thermo Fisher Scientific), with protein and chemical contamination estimated through absorption ratios (A260/A280 and A260/A230). The initial RNA concentrations used for this analysis ranged from 20 to 99 ng/µL.

### NanoString^®^ gene expression analysis

The nCounter^®^ Pro gene detection system allows for the simultaneous analysis of up to 800 genes, accommodating a wide range of RNA concentrations (from 1 ng to 100 ng) and various source materials (including cell lysates, whole blood, paraffin-embedded tissues, saliva, and blood smears) in a single 30 µL reaction volume. The nCounter^®^ Human Inflammation Panel (NanoString, Bruker Spatial Biology, WA, USA; catalog #XT-CSO-HIN2-12) profiles 255 genes, including 249 inflammation-related human genes and six internal controls (Table S1). These genes encode proteins involved in various inflammatory pathways, including interleukins, RAS proteins, T-cell receptors, and TLRs. Hybridization with the respective code sets was conducted for 18 h, adhering to the manufacturer’s minimum hybridization time of 16 h.

### Ethical statement

This study was conducted in accordance with the Declaration of Helsinki and received approval from the Ethics Committee of the Medical University of Sofia (Approval No. 07/23.04.2024).

### Informed consent

Written informed consent was obtained from all participants prior to sample collection and data processing. Participants were informed of the study objectives, procedures, and their right to withdraw at any time without repercussions.

### Data privacy and confidentiality

All collected data were anonymized prior to analysis to ensure participant confidentiality.

### Statistical analysis

Statistical analyses were conducted using nSolver™ Analysis Software, version 4.0 (NanoString^®^ Technologies Inc., Bruker Spatial Biology, WA, USA). Gene expression was evaluated at the sample level, comparing ten patients with stage III/IV periodontitis to two healthy controls. Raw data files (RCC format) and corresponding probe library files (RLF format) for all 12 individuals were imported into the software for quality control, normalization, and differential expression analysis. To adjust for biological sample variation, the software utilized the geometric mean of six stably expressed housekeeping genes (*CLTC, GAPDH, GUSB, HPRT1, PGK1,* and *TUBB*), as listed in Table S1. The mean percentage of probes exceeding the threshold across samples was 97.55% post-normalization, with a mean mRNA positive normalization factor of 1.008 (range: 0.89–1.26). All samples passed quality control and were included in the final analysis.

Between-group comparisons were carried out using the nSolver™ Analysis module, which employs a generalized linear model with unequal variance estimation for normalized count data. Raw *P* values from this model were adjusted for multiple testing using the Benjamini–Hochberg false discovery rate (FDR), with genes exhibiting FDR-adjusted *P* < 0.05 deemed statistically significant. The resulting normalized expression data were log_2_-transformed and exported as gene count tables for further analyses. Genes with mean normalized counts below 20 across all samples were excluded from statistical evaluation to reduce the impact of low-abundance transcripts, resulting in a final dataset of 89 genes (Table S2).

**Figure 1.**
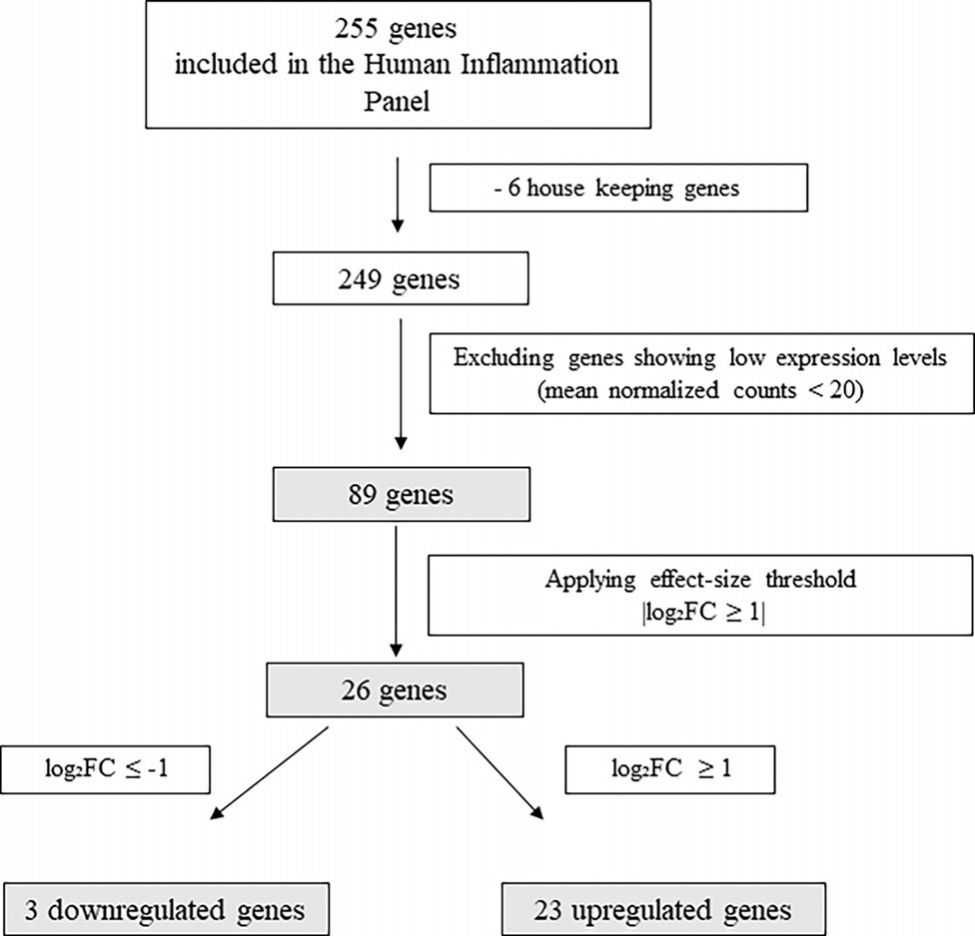
Flowchart illustrating gene filtering and differential expression based on log_2_ fold change.

**Figure 2.**
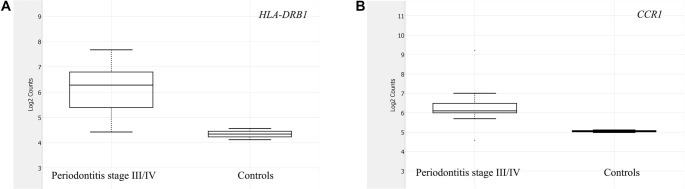
**Box plot analysis of gene expression in individuals with stage III/IV periodontitis (*n* ═ 10) compared to periodontally healthy controls (*n* ═ 2).** (A) Expression of *HLA-DRB1*; (B) Expression of *CCR1*. Expression values are presented as log_2_-normalized counts, derived from NanoString nSolver normalization.

**Figure 3.**
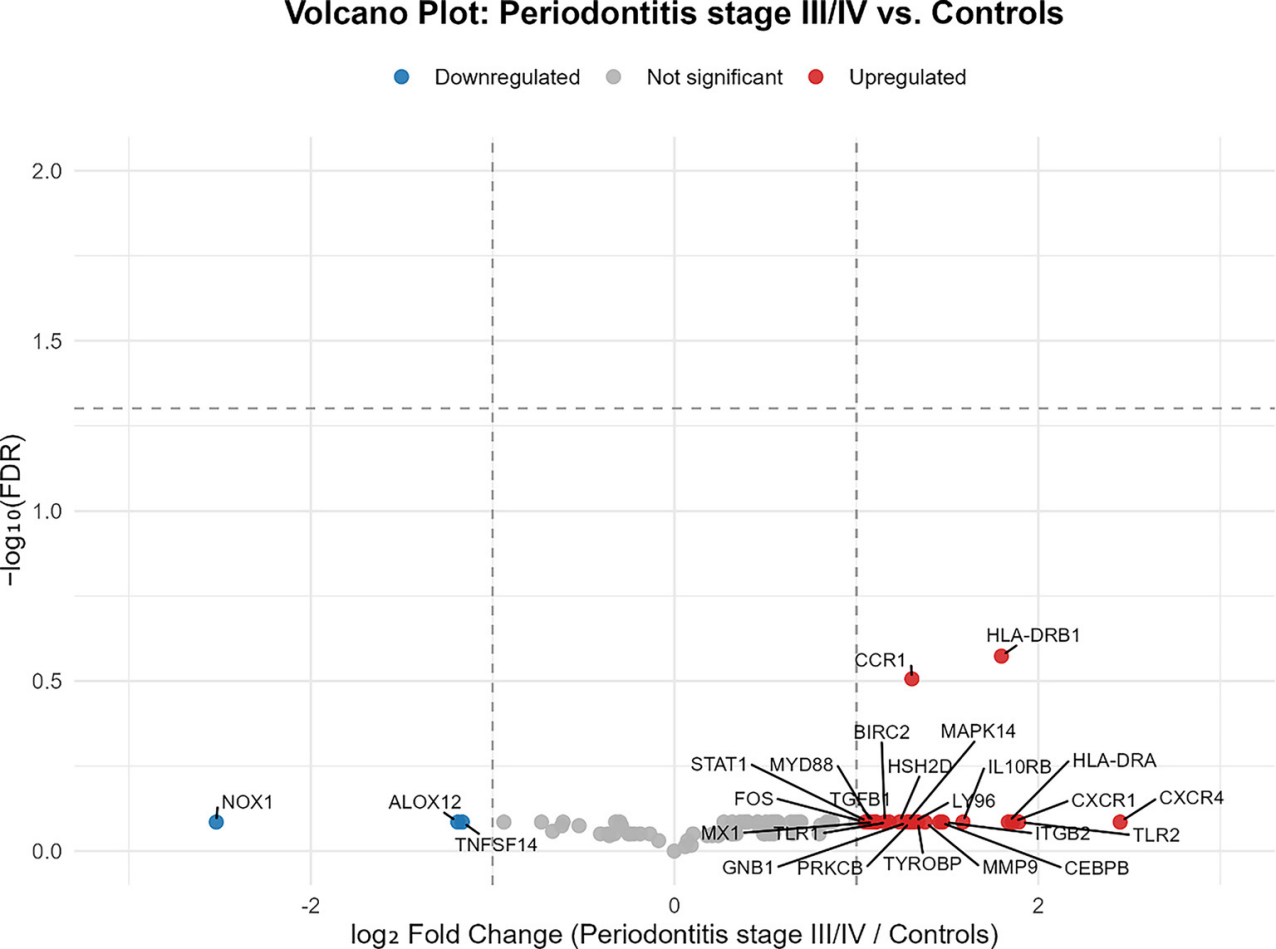
**Volcano plot illustrating differential gene expression in saliva samples from patients with severe periodontitis (*n* ═ 10) compared to periodontally healthy controls (*n* ═ 2).** Each point corresponds to an individual gene, plotted based on its log_2_ fold change (*x*-axis) and –log_10_ false discovery rate (FDR) (*y*-axis). Red points denote genes with log_2_ fold change > 1 (upregulated), blue points represent genes with log_2_ fold change < –1 (downregulated), and grey points indicate genes with smaller effect sizes.

Log2 fold changes (log_2_FC) and their 95% confidence intervals (CIs) were calculated in R (version 4.5.1) based on group means and standard deviations on the log2 scale to quantify effect-size uncertainty. Descriptive statistics were computed for all analyzed genes, and the workflow for gene filtering and identification of differentially expressed transcripts is illustrated in Figure 1. Expression differences were subsequently visualized using box plots (Figure 2) that highlight genes with the most significant up-regulation in the advanced periodontitis group, along with a volcano plot (Figure 3).

## Results

Following the gene expression analysis of 249 immune-related human genes in the nCounter Inflammation Panel, genes with low expression levels (mean normalized counts < 20) were excluded, yielding a final dataset of 89 genes for comparative analysis. Among these, 26 genes (29.2%) met a predefined effect-size threshold (|log_2_FC|≥ 1), comprising 23 upregulated (log_2_FC > 1) and 3 downregulated (log_2_FC < −1), as summarized in Figure 1.

Of the 26 genes with |log_2_FC|≥ 1 listed in Table 2, the majority were upregulated in patients with advanced periodontitis (23/26). This group included antigen-presentation and innate immune genes such as *HLA-DRB1, HLA-DRA, TLR2, LY96, MYD88, STAT1,* and *CXCR1/4*, along with transcripts involved in matrix remodeling and signaling (*MMP9, TGFB1, ITGB2, MAPK14, FOS*). Among the upregulated genes, *HLA-DRB1* (*P* ═ 0.003) and *CCR1* (*P* ═ 0.007) exhibited the lowest unadjusted *P* values. The three downregulated genes (log_2_FC < −1) were *ALOX12, NOX1,* and *TNFSF14*, none of which reached nominal significance. After correction for multiple testing using the Benjamini–Hochberg FDR method, no genes remained statistically significant, likely due to the limited sample size; thus, these results should be interpreted as exploratory. Nevertheless, the overall pattern of changes aligns with biological pathways implicated in severe periodontitis.

**Table 2:** Differentially expressed genes with |log_2_FC| ≥ 1 in patients with stage III/IV periodontitis compared to controls

**Gene**	**Transcript**	**Periodontitis** **(mean ± SD)**	**Control** **(mean ± SD)**	**Fold change (P/C)**	**log_2_FC**	**95% CI (lower)**	**95% CI (upper)**	***P* value**	**FDR adjusted *P* value**
*Upregulated genes*									
*HLA-DRB1*	NM_002124.1	70.35 ± 59.2	20.24 ± 4.45	3.48	1.8	0.93	2.67	0.003	0.267
*CCR1*	NM_001295.2	81.67 ± 173.38	33.05 ± 2.26	2.47	1.31	--0.6	3.21	0.007	0.312
*IL10RB*	NM_000628.3	65.62 ± 31.47	21.86 ± 7.27	3.0	1.59	0.79	2.38	0.052	0.822
*HLA-DRA*	NM_019111.3	102.13 ± 121.3	28.62 ± 21.77	3.57	1.84	--0.02	3.69	0.173	0.822
*CXCR4*	NM_003467.2	174.85 ± 128.3	32.0 ± 30.76	5.46	2.45	0.42	4.48	0.196	0.822
*BIRC2*	NM_001166.3	52.09 ± 17.79	23.37 ± 10.11	2.23	1.16	0.24	2.07	0.197	0.822
*TLR2*	NM_003264.3	110.61 ± 77.69	29.79 ± 24.74	3.71	1.89	0.12	3.67	0.226	0.822
*LY96*	NM_015364.2	129.92 ± 72.75	52.25 ± 31.77	2.49	1.31	--0.0	2.63	0.244	0.822
*MAPK14*	NM_001315.1	126.82 ± 49.4	52.25 ± 31.77	2.43	1.28	0.01	2.54	0.256	0.822
*CEBPB*	NM_005194.2	612.54 ± 181.91	222.91 ± 159.34	2.75	1.46	0.0	2.91	0.268	0.822
*MMP9*	NM_004994.2	71.2 ± 25.31	27.4 ± 18.81	2.6	1.38	--0.03	2.79	0.270	0.822
*TYROBP*	NM_003332.3	650.9 ± 362.42	257.57 ± 195.89	2.53	1.34	--0.26	2.94	0.289	0.822
*MX1*	NM_002462.2	120.86 ± 515.92	56.03 ± 40.57	2.16	1.11	--2.97	5.19	0.312	0.822
*MYD88*	NM_002468.3	118.64 ± 94.08	56.03 ± 40.57	2.12	1.08	--0.53	2.69	0.327	0.822
*STAT1*	NM_007315.2	59.97 ± 55.4	28.62 ± 21.77	2.1	1.07	--0.66	2.8	0.360	0.822
*CXCR1*	NM_000634.2	302.66 ± 195.89	83.44 ± 127.04	3.63	1.86	--1.24	4.96	0.373	0.822
*ITGB2*	NM_000211.2	197.49 ± 126.5	71.07 ± 82.98	2.78	1.47	--0.93	3.88	0.374	0.822
*TGFB1*	NM_000660.3	66.29 ± 41.17	30.91 ± 27.74	2.14	1.1	--0.78	2.98	0.396	0.822
*GNB1*	NM_002074.3	87.86 ± 34.44	36.01 ± 43.04	2.44	1.29	--1.13	3.7	0.432	0.822
*TLR1*	NM_003263.3	306.5 ± 144.78	135.25 ± 144.39	2.27	1.18	--1.0	3.36	0.430	0.822
*FOS*	NM_005252.2	5185.93 ± 2304.69	2508.44 ± 2375.69	2.07	1.05	--0.89	2.98	0.432	0.822
*HSH2D*	NM_032855.2	87.12 ± 57.04	36.95 ± 46.15	2.36	1.24	--1.33	3.8	0.453	0.822
*PRKCB*	NM_212535.1	95.22 ± 30.82	38.75 ± 52.42	2.46	1.3	--1.42	4.02	0.462	0.822
*Downregulated genes*									
*ALOX12*	NM_000697.1	20.46 ± 97.79	46.73 ± 20.23	--2.28	--1.19	--5.55	3.17	0.139	0.822
*NOX1*	NM_007052.4	20.12 ± 45.54	115.36 ± 123.62	--5.73	--2.52	--5.47	0.43	0.173	0.822
*TNFSF14*	NM_003807.2	21.46 ± 10.32	48.17 ± 23.09	--2.24	--1.17	--2.22	--0.12	0.202	0.822

Data are presented as mean ± SD of normalized gene expression for patients with stage III/IV periodontitis compared to periodontally healthy controls. The fold change is calculated as the ratio of periodontitis to control, with log_2_FC defined as log_2_(Fold change). The 95% confidence intervals (CIs) correspond to log_2_FC estimates derived from group means and variability, as calculated using R software. *P* values were obtained from the NanoString nSolver™ Analysis Software, utilizing background-corrected normalized counts and adjusted for multiple comparisons using the Benjamini–Hochberg method to control for false discovery rate (FDR).

The box plots in Figure 2 depict the expression distributions of *HLA-DRB1* and *CCR1* in patients with stage III/IV periodontitis compared to periodontally healthy controls. These genes were selected due to their significant log_2_ fold changes and the lowest unadjusted *P* values among the upregulated transcripts. The volcano plot in Figure 3 illustrates all 89 analyzed genes according to their log_2_FC and --log10(FDR), highlighting both the direction and strength of expression differences between groups. Collectively, these plots demonstrate the upregulation of *HLA-DRB1* and *CCR1* in advanced periodontitis, supporting their potential as exploratory salivary biomarker candidates for validation in larger cohorts.

## Discussion

Periodontitis is characterized by an intensified immune response. Our study provides evidence for the upregulation of several genes in individuals with severe periodontitis, confirming findings reported in previous studies. Among the most highly expressed genes, *HLA-DRB1* plays a pivotal role in antigen presentation and adaptive immunity. *HLA-DRB1* encodes an MHC class II molecule essential for presenting bacterial peptides to CD4^+^ T helper cells, thereby initiating and amplifying adaptive immune responses. Elevated expression of *HLA-DRB1* has been consistently observed in inflamed gingival tissues, reflecting enhanced immune recognition of periodontal pathogens such as *Porphyromonas gingivalis* and *Tannerella forsythia* [12]. Increased *HLA-DRB1* expression may enhance T-cell activation, recruiting additional immune cells to periodontal tissues and exacerbating local inflammation. Furthermore, specific *HLA-DRB1* alleles (such as _DRB1_03, *04,*14, and *15) are hypothesized to correlate with aggressive generalized periodontitis, suggesting that hyperactive antigen presentation could contribute to exaggerated immune responses and alveolar bone destruction. Chronic upregulation of *HLA-DRB1* in the context of persistent bacterial biofilm and IFN-γ-mediated signaling may drive excessive adaptive immune responses, promoting tissue damage and disease progression. In summary, the upregulation of *HLA-DRB1* in severe periodontitis reflects a biologically significant mechanism whereby antigen-presenting cells continuously stimulate CD4^+^ T cells, sustaining a Th1-dominated inflammatory environment that contributes to periodontal tissue destruction [13].

Conversely, *CCR1* is a chemokine receptor predominantly expressed on monocytes, neutrophils, and T cells, facilitating their migration to sites of inflammation in response to ligands such as CCL3 and CCL5. Its involvement in periodontitis has been highlighted in experimental models; for instance, Repeke et al. (2010) demonstrated the synergistic action of chemokines CCL3, CCL4, and CCL5 with CCR1 in promoting gingival inflammation and subsequent alveolar bone resorption [14]. High *CCR1* expression may lead to excessive recruitment of monocytes, macrophages, and T cells into periodontal tissues, thereby amplifying local inflammation through the release of pro-inflammatory cytokines such as TNF-α and IL-1β, ultimately contributing to tissue degradation and bone loss. Conversely, the absence or suppression of *CCR1* has been shown to reduce lymphocyte activation and alveolar bone destruction, indicating its potential as a therapeutic target to limit inflammatory cell infiltration [15]. Szczepaniak et al. [16] further reported elevated *CCR1* expression (along with CCR3 and CCR5) on T cells infiltrating inflamed periodontal sites, proposing that oral inflammation could propagate systemically, leading to organ damage, including cardiomyopathy, vascular injury, and hypertension. Injured tissues often upregulate *CCR1*, alongside other chemokines like CCR2, CXCR5, CCR5, and inflammatory mediators such as IL-8, TNF-α, CCL2, IL-6, and IL-1β, driving chronic, self-perpetuating inflammation that exacerbates periodontal tissue destruction [17, 18]. Interestingly, de Vries et al. described genotypes potentially protective against the disease—mice lacking *Tlr2/Tlr4* and *Ccr1/Ccr5* show a reduced inflammatory response. The overexpression of *CCR1* in severe periodontitis likely contributes to continuous immune cell recruitment and sustained local inflammation, establishing a chronic inflammatory environment that underpins tissue damage and alveolar bone loss.

In conclusion, the upregulation of *HLA-DRB1* and *CCR1* in stage III/IV periodontitis may reflect complementary mechanisms in disease pathogenesis: enhanced antigen presentation driving adaptive immunity and amplified leukocyte recruitment sustaining chronic inflammation. Although exploratory, these findings provide biologically plausible links to disease progression and identify *HLA-DRB1* and *CCR1* as promising biomarker candidates for further validation.

Additionally, Table 2 illustrates genes associated with known pathways implicated in periodontal pathogenesis. Genes related to TLR/MYD88-NF-κB innate sensing (*TLR2, LY96/MD-2, MYD88*) are consistent with the detection of dysbiotic biofilms by gingival and periodontal-ligament cells, activating nuclear factor kappa-light-chain-enhancer of activated B cells (NF-κB)-driven inflammatory pathways in periodontitis [19, 20]. Concurrently, chemokine-guided leukocyte recruitment is represented by *CCR1, CXCR1, CXCR4*, and *ITGB2*; experimental blockade or deficiency of *CCR1* reduces inflammatory cell influx and alveolar bone loss, CXCL12/CXCR4 signaling is detected in human gingival tissues, and defects in *ITGB2* are associated with severe, early-onset periodontitis [21–23]. Signals of antigen presentation (MHC-II) are represented by *HLA-DRA* and *HLA-DRB1*, consistent with reported associations of *HLA-DRB1* with susceptibility and antigen-presenting cell (APC)-mediated T-cell priming in diseased tissues [12]. Extracellular matrix remodeling is suggested by MMP9, *TGFB1,* and *ITGB2*; *MMP-9* is frequently elevated in periodontal samples and correlates with connective tissue breakdown, while *TGF-β1* is consistently increased in gingival tissue and crevicular fluid, modulating wound healing and fibrotic processes in periodontitis [24, 25]. Conversely, downregulated oxidative/lipid-mediator genes (*NOX1, ALOX12*) align with the well-documented redox component of the disease and reports of altered lipoxygenase expression in gingival tissue from patients with periodontitis [26, 27]. The identified pathway-associated gene alterations are biologically coherent with current models of periodontal inflammation and tissue destruction and warrant validation in larger patient cohorts.

### Limitations of the study

A limitation of our study is the relatively small patient cohort, which is restricted by the 12-sample capacity of the NanoString nCounter cartridge. This limited sample size reduces statistical power, contributing to the absence of FDR-significant findings. Nonetheless, the nCounter system allows for the simultaneous profiling of nearly 250 genes with high precision, minimizing biases associated with RT-PCR-based approaches that necessitate separate cDNA synthesis for each target. Although none of the genes achieved significance after FDR correction, the observed expression changes indicated biologically relevant trends consistent with the pathogenetic mechanisms of severe periodontitis. Additionally, the potential influence of confounding variables such as age, sex, and smoking status represents another limitation. Future studies with larger cohorts should incorporate these covariates into multivariable statistical models to better delineate disease-specific effects and enhance the generalizability of the findings. Despite these limitations, the high analytical sensitivity and accuracy of the NanoString system facilitate the detection of subtle yet biologically meaningful expression trends, providing a foundation for future validation of candidate molecular markers in severe periodontitis.

## Conclusion

In conclusion, our study identified *HLA-DRB1* and *CCR1* as the most prominently upregulated immune-related genes in patients with stage III/IV periodontitis. While their *P* values were significant in unadjusted analyses, statistical significance was not retained after FDR correction; nevertheless, these changes reflect biologically meaningful trends consistent with the known pathogenetic mechanisms of the disease. Specifically, the upregulation of HLA-DRB1 likely enhances antigen presentation to CD4^+^ T cells, driving hyperactive adaptive immune responses, while CCR1 overexpression may facilitate the recruitment of monocytes, macrophages, and T cells, thereby sustaining local inflammation and tissue destruction. These findings underscore key molecular pathways underlying severe periodontitis and present candidate genes for future investigation as potential salivary biomarkers or targets for immunomodulatory therapy.

## Supplemental data

Supplemental data are available at the following link: https://www.bjbms.org/ojs/index.php/bjbms/article/view/13313/4071.

## Data Availability

The datasets generated and analyzed during the current study are not publicly available due to privacy and ethical restrictions but are available from the corresponding author upon reasonable request.
